# Real-Time PCR Differential Detection of *Neorickettsia findlayensis* and *N. risticii* in Cases of Potomac Horse Fever

**DOI:** 10.1128/jcm.00250-22

**Published:** 2022-06-13

**Authors:** Khemraj Budachetri, Mingqun Lin, Qi Yan, Rory C. Chien, Laura D. Hostnik, Gillian Haanen, Mathilde Leclère, Warren Waybright, John D. Baird, Luis G. Arroyo, Yasuko Rikihisa

**Affiliations:** a Laboratory of Molecular, Cellular, and Environmental Rickettsiology, Department of Veterinary Biosciences, College of Veterinary Medicine, Infectious Diseases Institute, The Ohio State University, Columbus, Ohio, USA; b Veterinary Clinical Sciences, The Ohio State University, Columbus, Ohio, USA; c Moore Equine Veterinary Centre, Rocky View County, Alberta, Canada; d Department of Clinical Sciences, Faculty of Veterinary Medicine, University of Montréal, St. Hyacinthe, Québec, Canada; e Service Vétérinaire Ambulatoire, St-Lazare, Québec, Canada; f Department of Clinical Studies, Ontario Veterinary College, University of Guelph, Guelph, Ontario, Canada; Jockey Club College of Veterinary Medicine

**Keywords:** *Neorickettsia findlayensis*, *N. risticii*, Potomac horse fever, real-time PCR, culture isolation, enterotyphlocolitis

## Abstract

Potomac horse fever (PHF) is an acute and potentially fatal enterotyphlocolitis of horses with clinical signs that include anorexia, fever, diarrhea, and laminitis. Its incidence is increasing despite a commercially available vaccine. PHF is caused by Neorickettsia risticii, and the recently rediscovered and classified *N. findlayensis*. PHF diagnosis is currently accomplished using serology or nested PCR. However, both methods cannot distinguish the two *Neorickettsia* species that cause PHF. Further, the current *N. risticii* real-time PCR test fails to detect *N. findlayensis.* Thus, in this study, two *Neorickettsia* species-specific real-time PCR assays based on *Neorickettsia ssa2* and a *Neorickettsia* genus-specific real-time PCR assay based on *Neorickettsia* 16S rRNA gene were developed. The *ssa2* real-time PCR tests differentiated *N. findlayensis* from *N. risticii* in the field samples for which infection with either species had been verified using multiple other molecular tests and culture isolation, and the 16S rRNA gene real-time PCR detected both *Neorickettsia* species in the samples. These tests were applied to new field culture isolates from three Canadian provinces (Alberta, Quebec, Ontario) and Ohio as well as archival DNA samples from suspected PHF cases to estimate the prevalence of *N. findlayensis* in different geographic regions. The results suggest that *N. findlayensis* frequently causes PHF in horses in Alberta and Quebec. The development of these tests will allow rapid, sensitive, and specific diagnosis of horses presenting with clinical signs of PHF. These tests will also enable rapid and targeted treatment and help develop broad-spectrum vaccines for PHF.

## INTRODUCTION

Potomac horse fever (PHF) is an acute and potentially fatal enterotyphlocolitis of horses characterized by fever, lethargy, anorexia, dehydration, diarrhea, laminitis, and occasional abortion (1). PHF was originally described in the early 1980s as a new disease (“acute equine diarrhea syndrome”) on farms along the Potomac River in Virginia and Maryland. Since then, PHF has been diagnosed throughout the USA and several provinces in Canada and has been occasionally identified in Brazil, Uruguay, and Europe (1–5). In the early 1980s, electron microscopy analyses of samples from horses revealed distinct rickettsia-like bacteria mostly in the wall of the large colon with clinical signs of PHF (6, 7). In parallel, a rickettsial organism was isolated from the peripheral blood mononuclear cells of affected horses using the U937 human monocyte cell line (8, 9) or primary canine blood monocytes (10) and was named Ehrlichia risticii (11). Later, the bacterium was reclassified as Neorickettsia risticii (12). The most effective treatment for PHF is administration of oxytetracycline during the early stages of the disease (1). PHF typically occurs in the warm-weather months of late spring to early fall (1). Trematodes are natural reservoirs and vectors of *N. risticii*. Horses are infected by accidental ingestion of *N. risticii-*infected trematodes. Koch’s postulates were fulfilled following the oral administration of *N. risticii-*infected trematodes, which are endoparasites of aquatic insects (e.g., mayflies and caddisflies) to horses. *N. risticii* was culture isolated from blood mononuclear cells of horses that developed PHF (13, 14). Importantly, PHF occurs despite horses being vaccinated with a commercially available vaccine (*N. risticii*–based bacterin). This lack of protection is likely attributable to insufficient immunological response and various antigenic variants (15–17).

From 1984 to 2020, it was assumed that only *N. risticii* causes PHF, despite the finding that a molecularly and antigenically unique *Neorickettsia* sp. named 081 was cultured from a blood sample from a horse with PHF in Findlay, Ohio in 1991 (15, 18). *Neorickettsia* sp. 081 was recently classified as a new species named *N. findlayensis* along with two other new strains cultured from blood samples from horses with PHF in Ontario, Canada. This was based on whole genome sequencing of the type-strain Fin17 and by experimental infection that fulfilled Koch’s postulates (19, 20). Cell-culture isolation of *Neorickettsia* spp. from horses suffering with PHF provides a highly sensitive and definitive diagnosis (19, 21). However, owing to technical, economic, and time constraints, cell-culture isolation has succeeded only in a few laboratories in the USA (9, 10, 15, 19–22). Instead, PHF diagnosis is mainly accomplished by nested PCR that detects both *N. risticii* and *N. findlayensis* (19, 21) or *N. risticii* real-time PCR (quantitative PCR) (23) (both tests are based on the *Neorickettsia* 16S rRNA sequence) or serology (indirect immunofluorescence microscopy using *N. risticii* or *N. findlayensis*–infected P388D_1_ cells as antigens) (19, 21, 24). However, these methods cannot distinguish the two *Neorickettsia* species that cause PHF. Further, the current *N. risticii* real-time PCR test based on 16S rRNA gene fails to detect *N. findlayensis*, giving false negative test result (19). In addition, antibody tests cannot distinguish current from previous infection/exposure or vaccination, and the nested PCR test is cumbersome for routine clinical diagnosis which requires rapid turnover time. Thus, in this study, by comparing the genome sequences of *N. findlayensis* and *N. risticii* (19, 25) and other known *Neorickettsia* spp. (26–28), we developed a simple and rapid diagnostic method to distinguish *N. findlayensis* from *N. risticii* in addition to a method that detects both *Neorickettsia* spp. using well-defined clinical specimens. These tests were applied to the new field culture isolates from three Canadian provinces (Alberta, Quebec, Ontario) and OH, USA as well as archival DNA samples from suspect PHF cases to estimate the prevalence of *N. findlayensis* in different geographic regions. The results suggest that *N. findlayensis* frequently causes PHF in horses in Quebec and Alberta, encouraging future epidemiologic and ecological studies. These new rapid diagnostic methods will improve the current laboratory diagnosis of PHF.

## MATERIALS AND METHODS

### Study design.

First, new primers were designed for *Neorickettsia* species-specific and genus-specific real-time PCR assays. Second, validity of these real-time PCR assays was experimentally tested using known *N. findlayensis-* and *N. risticii*-positive clinical specimens which are culture-positive, *Neorickettsia* 16S rRNA nested PCR-positive, and multiple *Neorickettsia* sequence analysis-positive (19). Third, the new real-time PCR methods were applied to new clinical specimens including those from new geographic regions, which were culture-positive and *Neorickettsia* 16S rRNA nested PCR-positive, but *Neorickettsia* species unknown. Lastly, the new real-time PCR methods were applied to PHF-suspected clinical specimens, which were *Neorickettsia* 16S rRNA nested PCR-positive or -negative but had no other test results available.

### Real-time PCR primer design, real-time PCR.

To design real-time PCR targets and primers for *N. findlayensis* and *N. risticii* differential assay, we first compared whole genome sequences of all *Neorickettsia* species (*N. findlayensis* Fin17 - GenBank accession no. NZ_CP047224; *N. risticii* Illinois - NC_013009.1; *N. sennetsu* Miyayama - NC_007798.1; *N. helminthoeca* Oregon - NZ_CP007481.1) (19, 25, 26, 29) by synteny analysis using MUMmer program (30). For phylogenetic analysis of 16S rRNA gene and SSAs, we also included *Neorickettsia* sp. SF (*Stellantchasmus falcatus*) stain sequences (27, 31) by aligning nucleotide or protein sequences using CLUSTAL Omega (32) in MegAlign Pro program of DNAStar Lasergene 17 (Madison, WI). To estimate confidence levels in phylogenetic analysis, bootstrap values for 1,000 replicates were calculated by Maximum likelihood using RAxML option by MegAlign Pro program (DNAStar). The sequence distance is calculated by dividing the numbers of nucleotide or amino acid differences by the total numbers of nucleotides and amino acids among homologous sequences. Furthermore, *N. risticii* and *N. findlayensis* strain-variable and -conserved sequences of clinical specimens (15, 19, 33) were taken into consideration. Based on these analyses, two pairs of primers unique to each *Neorickettsia* sp.: Nfin_Ssa2F/Nfin_Ssa2R (for *N. findlayensis*) and Nris_Ssa2F/Nris_Ssa2R (for *N. risticii*) were designed (Table 1). Although the 16S rRNA genes from all *Neorickettsia* spp. are quite conserved, currently used *N. risticii* 16S rRNA gene-based real-time PCR (23) cannot detect *N. findlayensis* (19). To develop a pan-*Neorickettsia* spp. real-time PCR assay, 16S rRNA genes of all *Neorickettsia* spp. including *N. findlayensis* and *N. risticii* were aligned, and primers: Neorick16S_F/Neorick16S_R were designed to amplify 16S rRNA genes of all *Neorickettsia* spp. except *N. helminthoeca* (Table 1). All primer sequences were blasted against the entire GenBank database to assure the specificity.

**TABLE 1:** Primer sequences for *Neorickettsia findlayensis* and *N. risticii*

Target gene	Primer name	Direction	Sequence (5′–3′)	References
*N. findlayensis* ssa2	Nfin_Ssa2F	Forward	GAAAACGGCGCTAAAGATAAGG	This study
	Nfin_Ssa2R	Reverse	TCCTTAGCTGTATCATTCTTCAGTACC	This study
*N. risticii* ssa2	Nris_Ssa2F	Forward	CGAAAACAGCGGCAGAGACA	This study
	Nris_Ssa2R	Reverse	CTTTTGTTGCATCGGTGAACAGG	This study
*Neorickettsia* 16S rRNA (qPCR)	Neorick16S_F	Forward	GTGTGAAATCCTTGGGCTTAACC	This study
	Neorick16S_R	Reverse	AACACTCATCGTTTACAGCGTGG	This study
*Neorickettsia* 16S rRNA (nested PCR, 1^st^ round)	ER-5-3	Forward	ATTTGAGAGTTTGATCCTGG	Chaichanasiriwithaya et al., (15)
	ER-3-2	Reverse	GTTTTAAATGCAGTTCTTGG	Chaichanasiriwithaya et al., (15)
*Neorickettsia* 16S rRNA (nested PCR, 2^nd^ round)	Eris-1	Forward	GGAATCAGGGCTGCTTGCAGCCT	Mott et al., (21)
	Eris-2	Reverse	TGTGGGTACCGTCATTATCTTCCCCA	Mott et al., (21)

DNA was purified directly from the buffy coats of the clinical horse blood samples or from *Neorickettsia* in the P388D_1_ cell cultures using the DNeasy blood and tissue kit (Qiagen, Valencia, CA, USA). All real-time PCR assays were performed using extracted DNA (approximately 150 ng) as the template, Maxima SYBR green/ROX qPCR Master Mix (2×; Thermo Fisher Scientific, Waltham, MA), and 250 nM (final concentration) of each primer in a 20-μL reaction mixture with a thermal cycle of 95°C for 30 sec followed by 35 cycles of 95°C for 30 sec, 58°C for 1 min, and 68°C for 30 sec in a Mx3000P Multiplex Quantitative PCR system (Stratagene, La Jolla, CA). All real-time PCR tests were conducted in parallel with positive control templates (*N. findlayensis* Fin17 or *N. risticii* PA-1 DNA) and negative controls (buffer only).

To determine sensitivities of the real-time PCR assays, the PCR products of *N. findlayensis* and *N. risticii ssa2* and the 16S rRNA gene were cloned into the TOPO vector (Zero Blunt TOPO PCR Cloning kit; Thermo Fisher Scientific), and the plasmids were purified using the GeneJET Plasmid Miniprep kit (Thermo Fisher Scientific). The plasmids were validated by sequencing. Using 10-fold serially diluted plasmids as the template (from 10^8^ copies/μL of plasmid to 10^1^ copies/μL), the minimum copy number of the plasmid detectable in each real-time PCR was estimated as (X × 6.0221 × 10^23^ molecules/mole) divided by (N × 660 g/mole) × 10^9^, where X is the number of nanograms of the plasmid DNA and N is the size (in bp) of the plasmid with the insert.

### Sequencing PCR products.

Approximately 300 ng DNA template was added to 0.625-unit *Taq* DNA polymerase with 10× Standard *Taq* reaction buffer (New England BioLabs, Ipswich, MA), (2.5 μL), 2 mM MgCl_2_ (Thermo Scientific), 0.2 mM (each) dNTPs, with 400 nM (each) the appropriate primers in 25-μL reaction volume in a GeneAmp PCR System 9700 Thermal Cycler (Applied System, Foster City, CA). The PCR products were analyzed with a 1% agarose gel containing 0.5 μg/mL ethidium bromide and visualized under UV light (Amersham Imager 680 QC, GE Healthcare BioSciences, Marlborough, MA). The PCR products for species-specific *ssa2* were purified using the GeneJet PCR purification kit, and all 11 PCR products of newly isolated *Neorickettsia* spp. and *N. risticii* PA-1 were sequenced to validate the *ssa2* amplicons (176 bp for *N. findlayensis*, 149 bp for *N. risticii*) at The Ohio State University Comprehensive Cancer Center Genomics Shared Resource Facility.

### Nested PCR to detect *Neorickettsia* spp.

The Rikihisa Laboratory at The Ohio State University has been using a nested PCR method developed previously (21) using two primer pairs: the primers ER-5-3 and ER-3-2 were used in the first PCR, and Eris-1 and Eris-2 for the second PCR (Table 1). In the first reaction, approximately 300 ng DNA template was added to 0.625-unit *Taq* DNA polymerase with 10× Standard *Taq* reaction buffer, 2.5 mM MgCl_2_, 0.2 mM each dNTPs, and 400 nM (final concentration) each primer in a 25-μL reaction volume. In the nested reaction, 3 μL of the first reaction product was used as the template with the second pair of primers. Both steps of the nested PCR were run in a GeneAmp PCR System 9700 Thermal Cycler as follows: one cycle at 95°C for 5 min, 35 cycles of 95°C for 1 min, 60°C for 1 min, and 68°C for 1 min, followed by extension at 68°C for 7 min. For each set of reactions, a buffer control and positive control containing *N. risticii* PA-1 DNA were conducted in parallel. The amplicons were analyzed with a 1% agarose gel containing ethidium bromide and visualized under UV light using Amersham Imager 680 QC.

### PHF cases.

The PHF cases comprised newly cultured isolates from 11 horses diagnosed in 2018–2020. Molecular diagnostic testing for other common enteropathogens using a commercial equine diarrhea panel at IDEXX Laboratories, Inc. (West Sacramento, CA) was negative for Salmonella spp., Clostridium difficile toxin genes A and B, C. perfringens, and equine Coronavirus in these horses. Some *N. findlayensis* samples were tested by *N. risticii* real-time PCR at IDEXX Laboratories, and at the Animal Health Laboratory (AHL), University of Guelph.

### Culture isolation of *Neorickettsia*.

For each horse, approximately 50 mL of blood was collected into EDTA-coated tubes that were then transported within 48 h to the Rikihisa laboratory, The Ohio State University (Columbus, Ohio), for culture. Blood from each horse was centrifuged at 500 × *g* for 10 min to obtain the buffy coat, and the remaining red blood cells in the buffy coat were lysed with an ammonium chloride solution to yield peripheral blood leukocytes (15). The leukocyte preparations were individually inoculated into P388D_1_ cell preparations (15), which were then cultured in RPMI 1640 medium containing 5% fetal bovine serum (21). Samples of the cultured cells were examined weekly for signs of infection under a light microscope after Diff-Quik staining (15). When infection was detected, ~0.5 mL of the culture was harvested for the isolation of DNA that was then used in a nested PCR test directed to the *Neorickettsia* 16S rRNA gene to amplify a 382-bp sequence (21) (Table 1). At least 75% of each culture medium was replaced with fresh RPMI 1640 containing 5% fetal bovine serum weekly until infection was seen or the experiment was ended by freezing the infected cells.

### GenBank accession numbers.

GenBank accession numbers for the *N. findlayensis ssa2* sequences from horses Alb20, Cin20, Dom20, Zig20, Til20, and Bul20 are MZ161203 to MZ161206, OK491930, and OK491931, respectively. GenBank accession numbers for the *N. risticii ssa2* sequences from horses Bla20, Chu18, Oreo20, Rog18, Whi18, and PA-1 are OL362023 to OL362027, and OL657171, respectively.

## RESULTS

### Development of new real-time PCR methods.

**FIG 1:**
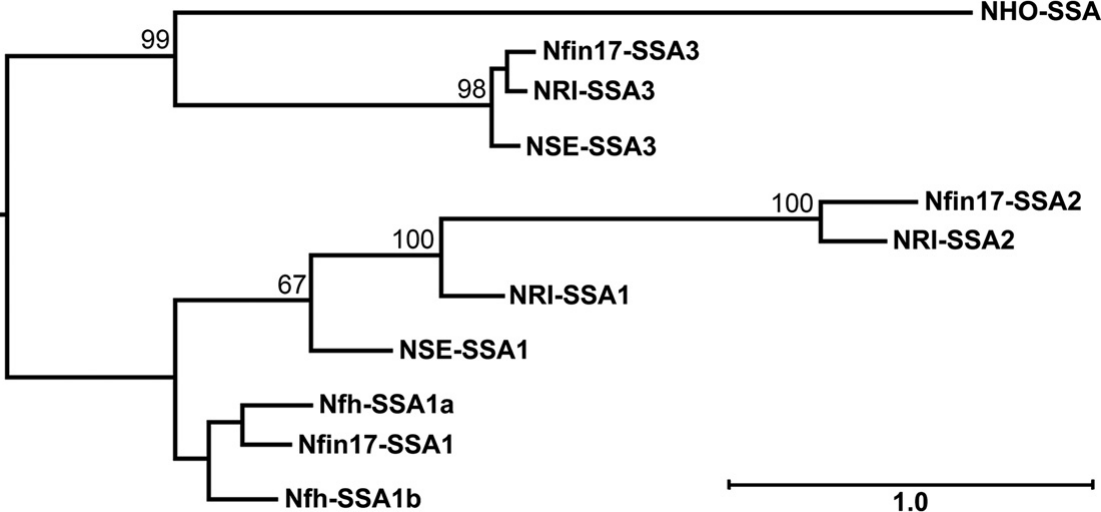
Phylogenetic analysis of *Neorickettsia* SSA proteins. *Neorickettsia* SSA proteins were aligned using Clustal Omega, and the phylogenetic tree was constructed with MegAlign Pro. Bootstrap values were calculated by maximum likelihood using RAxML options. The bar indicates sequence distances: the number of amino acid substitutions per site. *N. helminthoeca* encodes only one SSA protein (333 amino acid residues), whereas *Neorickettsia* sp. 179522 (endosymbiont of Fasciola hepatica) encodes two smaller SSA proteins (SSA1a: 197 residues, and SSA1b: 344 residues) that are most closely related to *N. findlayensis* SSA1. Abbreviations and NCBI accession numbers: Nfin17 (*N. findlayensis* Fin17) – SSA1: WP_160095937, SSA2: WP_160095941, SSA3, WP_160095943.1; NRI (*N. risticii* Illinois) – SSA1: WP_015816716, SSA2: WP_015816703, SSA3, WP_015816717; NSE (Neorickettsia sennetsu Miyayama) – SSA1: WP_011452276.1, SSA3: WP_011452279.1; NHO (*N. helminthoeca* Oregon) – SSA: WP_038560160; Nfh (*Neorickettsia* sp. 179522) – SSA1a: WP_067980232, SSA1b: WP_067980235.

Based on comparison of all available whole genome sequences of *Neorickettsia* species and other available *Neorickettsia* spp. sequences at GenBank, and *N. risticii* and *N. findlayensis* strain-variable and -conserved sequences of clinical specimens, the real-time PCR target and species-specific primers were designed. For example, P51 is a 51-kDa *Neorickettsia* outer membrane ß-barrel protein composed of 18 transmembrane segments which are *Neorickettsia* species conserved, and an variable extracellular domain containing nine loops (34). In particular the surface-exposed loop 2 of *p51* is highly variable among isolated *N. risticii* strains. Thus, using primers designed for the conserved flanking transmembrane domains it is possible to amplify the surface-exposed loop 2 of all *Neorickettsia* species and sequence the PCR product for initial confirmation and comparison of *Neorickettsia* isolates (27, 34, 35). However, high strain variability is not suitable to design species-specific real-time PCR primers within the surface-exposed loop 2. Another variable *Neorickettsia* genomic locus consists of two to three tandem *ssa* genes encoding “strain-specific antigens” (SSAs), as shown by whole genome synteny alignment (Fig. S1). Among them, *ssa1* DNA sequences are highly variable among *Neorickettsia* strains (19), which precludes the development of species-specific PCR tests. In addition, *ssa1* lacks the conserved sequences suitable for designing primers to amplify *ssa1* of all *N. risticii* or *N. findlayensis* strains. Although our previous study found *ssa3* is useful to distinguish *N. findlayensis* from *N. risticii* by regular PCR (19), extensive intramolecular base sequence repeats made it impossible to design real-time PCR primers. However, based on comparison of whole-genome sequences of all available *Neorickettsia* spp., only *N. findlayensis* and *N. risticii* have *ssa2* encoding “strain-specific antigen” 2 (Fig. 1). Although *Neorickettsia* sp. 179522 from Fasciola hepatica is most closely related to *N. findlayensis* based on 16S rRNA gene sequence comparison (Fig. S2) (19), and the assembled genome of *Neorickettsia* sp. 179522 contains two truncated *ssa1* genes, it lacks *ssa2* (Fig. 1). Alignment of the *ssa2* sequences of the *N. findlayensis* Fin17 strain (19) and the *N. risticii* Illinois strain (25) revealed unique sequence regions for each species (Fig. S3). Thus, we designed two sets of primer pairs based on *ssa2* to specifically amplify *N. findlayensis* and *N. risticii*, respectively, by real-time PCR (Table 1).

**FIG 2:**
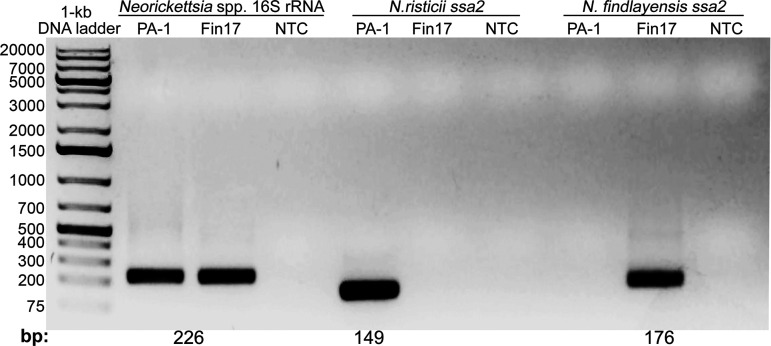
Real-time PCR products of *Neorickettsia* spp. 16S rRNA gene, and *ssa2* genes specific to *N. findlayensis* Fin17 and *N. risticii* PA-1. The PCR amplicons were electrophoresed on a 1% agarose gel, and the expected PCR product lengths are shown at the bottom. NTC, No template control.

Although the 16S rRNA genes from all *Neorickettsia* spp. are quite conserved, currently used *N. risticii* 16S rRNA gene-based real-time PCR (23) cannot detect *N. findlayensis* (19). Thus, we also designed a pair of real-time PCR primers based on the 16S rRNA gene (Table 1) that can detect all *Neorickettsia* spp. except *N. helminthoeca.* Fig. 2 shows the real-time PCR product sizes and specificity of *Neorickettsia ssa2*–based PCR using the *N. findlayensis* Fin17 and *N. risticii* PA-1 strains as templates (19). *N. risticii* Illinois and PA-1, a year 2000 isolate in Pennsylvania have identical 16S rRNA gene sequence (33, 34). The PCR product sequences of the *ssa2* gene of PA-1 was identical to *ssa2* of *N. risticii* Illinois type strain. Using serially diluted *N. findlayensis ssa2*, *N. risticii ssa2*, and *Neorickettsia* 16S rRNA gene–encoding plasmids, we determined that the real-time PCR primer pairs could detect as few as 21 copies of *ssa2* of *N. findlayensis*, 210 copies *ssa2* of *N. risticii*, and 24 copies of 16S rRNA gene, respectively.

### Specificity of the new real-time PCR of known clinical isolates.

Three culture-positive clinical samples of *N. findlayensis* (two from Ontario, Canada, and one from Ohio), and 10 culture-positive clinical samples of *N. risticii* previously analyzed by multigene sequence analysis (19) were tested by the real-time PCR primer pair. The *ssa2* real-time PCR tests detected all three *N. findlayensis* and all 10 *N. risticii* strains, respectively, and there was no cross-amplification between two *Neorickettsia* species (Table 2). Further, the new 16S rRNA gene–specific pan *Neorickettsia* real-time PCR test detected all three *N. findlayensis* and all 10 *N. risticii* strains of known culture-positive clinical specimens (Table 2). As negative control, 15 clinical samples that were negative by the *Neorickettsia* 16S rRNA gene–specific nested PCR test during 2020 and 2021 at the Ohio State University, were tested by *ssa2*-based PCR assays and the new 16S rRNA-based real-time PCR assay. All assays for these specimens were negative.

**TABLE 2:** Validation of *ssa2* real-time PCR assay using known *N. findlayensis* and *N. risticii* clinical isolates^*a*^

Horse ID^*c*^	NFIN^*b*^, verified^*c*^	NRI^*b*^, verified^*c*^	*Neorickettsia* spp. (nested PCR)^*c*^	*Neorickettsia* spp. (qPCR)^*d*^	NFIN (qPCR)^*d*^	NRI (qPCR)^*d*^
081 Ohio	+	−	+	+	+	−
Fin17	+	−	+	+	+	−
Tom16	+	−	+	+	+	−
May17	−	+	+	+	−	+
Luc17	−	+	+	+	−	+
Cup17	−	+	+	+	−	+
Lad17	−	+	+	+	−	+
Dun17	−	+	+	+	−	+
Jan17	−	+	+	+	−	+
Gab17	−	+	+	+	−	+
Dai17	−	+	+	+	−	+
Too16	−	+	+	+	−	+
Reg16	−	+	+	+	−	+

a +, positive; −, negative.

b NFIN, *N. findlayensis*; NRI, *N. risticii*.

c Teymournejad O, et al. 2020. (19)

d qPCR, real-time PCR (this study).

### Application of the new real-time PCR tests to new clinical isolates.

Next, these new real-time PCR tests were applied to three 2018 and eight 2020 culture isolates from horses with PHF. The real-time PCR results were also compared to those obtained from an established method using nested primers directed to the 16S rRNA gene (Table 3). The last two numbers in each horse ID indicate the year (2018 or 2020) of stable *Neorickettsia* species culture isolation. The horses resided near a river, stream, or lake in four geographic locations: Quebec, Alberta, and Ontario in Canada, and OH, USA (Table 3). The horses resided along the St. Lawrence River in Ontario and Quebec, along the Richelieu River in Quebec, near many lakes, rivers, and creeks, such as “Nose Creek” and Red Deer River near Calgary, Alberta, near Lake Simcoe in Ontario, Canada, and along the Tuscarawas River, Ohio. Near Calgary, Dom20 and Alb20 resided near many lakes. Zig20 resided near “Nose Creek” and small lakes, and Cin20 resided near Red Deer River, and many PHF cases from Alberta were previously reported based on serological test results (36). Table 3 presents data concerning the number of days each horse was observed to be sick by the owner before the attending veterinarian first examined the horse and collected blood samples, the clinical signs, and the vaccination status of each horse. The following clinical signs were recorded: lethargy, anorexia, fever, color of mucous membranes (buccal and conjunctival), nature of diarrhea [mild diarrhea (softer than normal), moderate (“cow-pie”), severe (watery, profuse, projectile)], and laminitis (hoof pain). The horses showed typical clinical signs of PHF, including fever (8/11), lethargy (11/11), anorexia (11/11), diarrhea (9/11), abnormal mucous membrane color (11/11), and laminitis (0/11) (Table 3). All 11 samples tested positive using both nested PCR for *Neorickettsia* spp. and *Neorickettsia* spp.–specific real-time PCR directed to the 16S rRNA gene (Table 3). At least eight of the 11 horses were not vaccinated for PHF. Using *ssa2*-directed real-time PCR, four of four (100%) *Neorickettsia* isolates from Alberta were identified as *N. findlayensis*, and two isolates from three cases (66%) from Quebec were identified as *N. findlayensis*. The remaining cases, i.e., three from Ontario, and one each from Quebec and Ohio, were identified as *N. risticii* (Table 3). The PCR product sequences of the *ssa2* gene of all six *N. findlayensis* isolates were identical to *ssa2* of Fin17 type strain (19) except for a single base-pair difference in Dom20. The PCR product sequences of the *ssa2* gene of all five *N. risticii* isolates were identical to *ssa2* of Illinois type strain (25), but distinct from those of *N. findlayensis* (GenBank accession Numbers are shown in the Material and Methods section). A previous study reported currently used *N. risticii*-specific real-time PCR (23) fails to detect culture-positive *N. findlayensis* in clinical specimens (19, 20). In the present study, blood samples of all four culture-positive *N. findlayensis* samples from Alberta were negative by IDEXX *N. risticii* real-time PCR test (Table 3). Out of total six culture-positive *N. findlayensis* strains, five were tested by *N. risticii* real-time PCR test at AHL-University of Guelph, and the results were all negative (Table 3).

**TABLE 3:** PCR test results of blood samples, region of origin, clinical information, vaccination status, and treatment outcome of horses from which *Neorickettsia* species were isolated in 2018 and 2020^*a*^

Horse ID^*b*^	*Neorickettsia* spp. (nested PCR)	*Neorickettsia* spp. (qPCR)	NFIN^*c*^ (qPCR)	NRI^*c*^ (qPCR)	NRI^*c*^ (qPCR) AHL	NRI^*c*^ (qPCR) IDEXX	Region	Age (yrs)	Stable at night	Sick (days)	PHF vaccinated	Lethargy	Anorexia	Fever	Diarrhea	Mucous membrane^*e*^
Whi18	+	+	−	+	NT^*d*^	NT	Ontario	14	No	0	Yes	Yes	Mild	Yes	Yes cow pie	Pale pink
Rog18	+	+	−	+	NT	NT	Ontario	14	No	0	Yes	Yes	Yes	Yes	No	Pink
Chu18	+	+	−	+	NT	NT	Ontario	7	No	5	No	Yes	Transient	Yes	Yes	Pale pink
Bul20	+	+	+	−	NT	NT	Quebec	5	Yes	1	No	Yes	Yes	Yes	Yes, cow pie	Pale pink
Bla20	+	+	−	+	NT	NT	Quebec	14	Yes	1	No	Yes	Yes	Yes	Yes	Pale pink
Til20	+	+	+	−	−	NT	Quebec	10	No	5	No	Yes (mild)	Yes	Yes	No	pale pink
Ore20	+	+	−	+	NT	NT	Ohio	21	Yes	4	Not known	Yes	Yes	No	Yes	Dark, pink, Toxic line
Alb20	+	+	+	−	−	−	Alberta	13	No	2	No	Yes	Yes	No	Yes profuse projectile	Dark pink, toxic line
Cin20	+	+	+	−	−	−	Alberta	4	No	4	No	Yes	Yes	No	Yes profuse projectile	Dark pink
Dom20	+	+	+	−	−	−	Alberta	9	No	3	No	Yes	Yes	Yes	Yes profuse projectile	Dark pink, toxic line
Zig20	+	+	+	−	−	−	Alberta	7	No	0	No	Yes	Yes	Yes	Yes mild	Dark pink

a +, positive; −, negative.

b The last two numbers in each horse ID indicate the year (2018 or 2020) of stable *Neorickettsia* species culture isolation.

c NFIN, *N. findlayensis;* NRI, *N. risticii*.

d NT, not tested.

e None of horses developed laminitis, and all had been treated and recovered.

### Application of the new real-time PCR to archival DNA samples from suspected clinical PHF cases.

The *ssa2* real-time PCR was applied to stored DNA of blood samples from horses suspected of having PHF in 2019 and 2020. Of 67 suspected PHF cases from Ontario, 33 tested positive by nested *Neorickettsia* 16S rRNA gene–based PCR (2 were identified as *N. findlayensis*, and 31 were identified as *N. risticii* by *ssa2* real-time PCR), and the remaining 34 were negative by all three PCR tests, implying 34 animals were not PHF cases (Table 4). Of 11 suspected PHF cases from The Ohio State University Veterinary Teaching Hospital from 2011 to 2020, 10 were positive for *Neorickettsia* by nested PCR directed to the 16S rRNA gene. All of the positive samples were identified as *N. risticii*, but not *N. findlayensis* by *ssa2* real-time PCRs (Table 4).

**TABLE 4:** Prevalence of *Neorickettsia* sp. (*N. findlayensis* and *N. risticii*) in archived blood DNA samples

Yr	Samples (*N*)	*NFIN*^a^ (*ssa2* qPCR)	*NRI*^*a*^ (*ssa2* qPCR)	*Neorickettsia* spp. nested PCR	Negative
2019 (Ontario, Canada)	26	1	7	8	18
2020 (Ontario, Canada)	41	1	24	25	16
Totals	67	2	31	33	34
2011–2020 Ohio	11	0	10	10	1

a NFIN, *N. findlayensis*; NRI, *N. risticii*.

## DISCUSSION

Currently there is no rapid method available to diagnose *N. findlayensis* infection. This study describes the development and validation of *N. findlayensis*–specific and *N. risticii*–specific real-time PCR tests directed to *ssa2*. Known *N. findlayensis* and *N. risticii*–culture-positive clinical specimens were used to validate the tests. Recent (19, 20) and current studies showed currently used *N. risticii* 16S rRNA gene–specific real-time PCR fails to detect *N. findlayensis* in clinical specimens. However, the new 16S rRNA gene–based *Neorickettsia* genus-specific real-time PCR developed in the present study detected *N. findlayensis* in all clinical specimens from diverse geographic regions in 1991–2020. Interestingly, there was no co-infection of two *Neorickettsia* species in any of PHF cases. This assay is expected to reduce false negative PCR diagnosis of PHF due to *N. findlayensis* infection. Moreover, compared with the classic 16S rRNA gene–based nested PCR, the newly developed pan-*Neorickettsia* real-time PCR is similarly effective in the molecular diagnosis of PHF. Using plasmids containing *ssa2*s or the 16S rRNA gene, the ability of these assays to detect *N. findlayensis*, *N. risticii* and the *Neorickettsia* genus are shown for the first time and informs us of the PCR detection threshold (PCR assay negative if bacterial numbers are below the threshold). The current result is expected to encourage future investigation to analyze PHF diagnostic sensitivities and specificities for the new real-time PCR tests with a larger number of clinical specimens.

**FIG 3:**
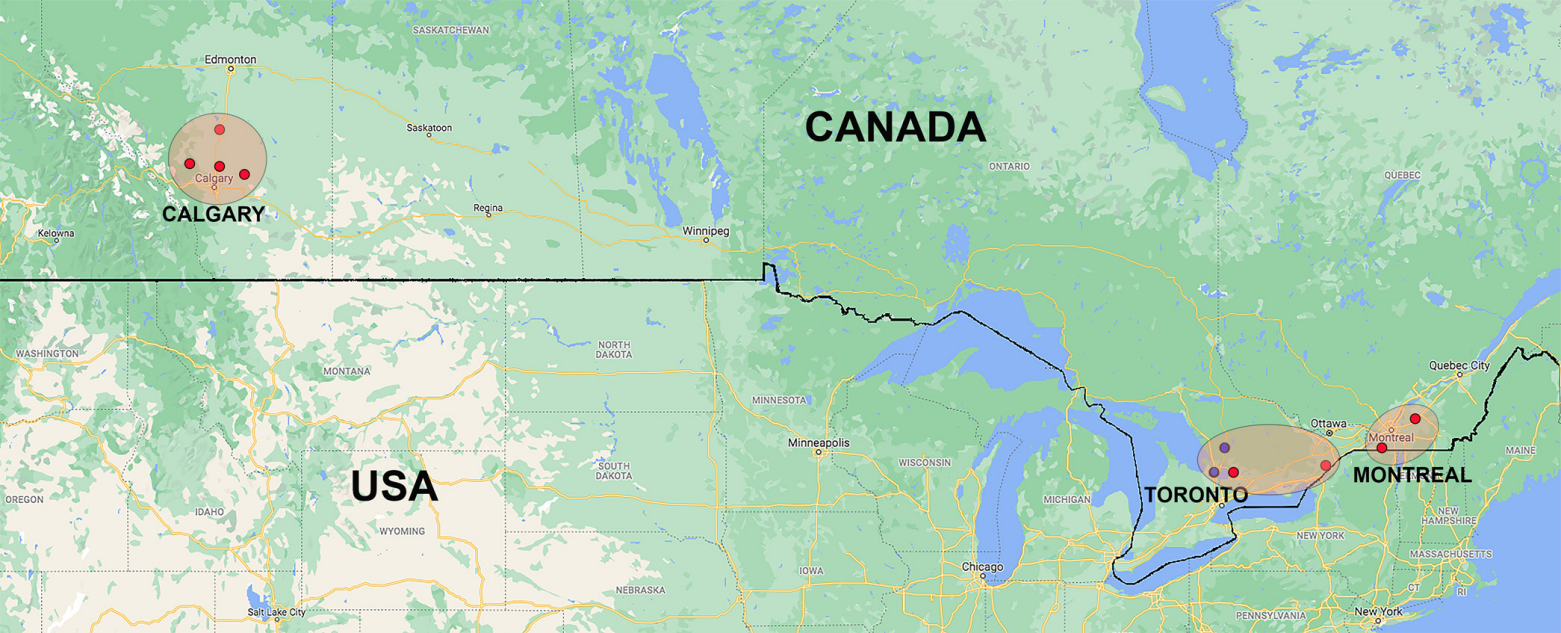
Geographic locations of *N. findlayensis-*infected horses with PHF. Red dots represent the geographic locations of clinical cases from which *N. findlayensis* was isolated from blood of horses and verified by real-time PCR: four in Calgary, Alberta and two in Montreal, Quebec from this study, and two in Toronto, Ontario were from previous study (19). Blue dots represent clinical cases from which *N. findlayensis* was detected by real-time PCR of blood specimens (two in Toronto, Ontario from this study).

The utility of the developed assays was evaluated with clinical isolates submitted from new geographic regions as well as with stored DNA samples from suspected PHF cases. Although we could assess only a limited number of culture-positive cases, the current study reveals that PHF caused by *N. findlayensis* infection is widespread in Canada, and this pathogen is potentially a major cause of PHF in certain geographic regions (Alberta and Quebec), but a minor cause of PHF in other geographic regions (Ontario and Ohio). Fig. 3 depicts geographic distribution of *N. findlayensis* in Canada confirmed by culture isolation and/or the current real-time PCR. Using both the *N. risticii* and *N. findlayensis* real-time tests directed to *ssa2*, the proportions of PHF caused by *N. findlayensis* in other geographic regions can be investigated. Combining these tests with the *Neorickettsia g*enus-specific real-time PCR test would facilitate the discovery of other *Neorickettsia* species that may cause PHF.

*Neorickettsia* spp. are Gram-negative obligatory intracellular bacteria of digenean trematode flukes, which are transmitted through all developmental stages of the trematodes and vertically through generations of trematodes (37–41). Several *Neorickettsia* spp. are horizontally transmitted from infected trematodes to humans, horses, and dogs and cause severe diseases in these accidental hosts (42). Thus, the geographic distribution and prevalence of PHF in areas where horses are raised likely corresponds to the distribution of *Neorickettsia* spp.–infected trematodes and the intermediate and definitive hosts of the trematodes. In the case of *N. risticii*, each developmental stage of the trematode species naturally infected with this bacterium in Pennsylvania was morphologically and molecularly identified as *Acanthatrium oregonense* (33). Big brown bats (Eptesicus fuscus) and little brown bats (Myotis lucifugus) are infected with *N. risticii* as confirmed by 16S rRNA nested PCR and *p51* (encoding the major outer membrane protein P51 of porin activity unique to *Neorickettsia* spp. (27, 43)) PCR-positive bat liver, spleen and/or blood samples, followed by sequencing the PCR products (37). The intestinal lumen of these bats harbors adult gravid trematodes infected with *N. risticii* and individual eggs isolated from them were also infected with *N. risticii* (37). It is of great interest to uncover the trematode species that carry *N. findlayensis* and hosts of trematodes in Canada, and whether the trematode’s natural hosts are infected.

The course of PHF is usually 5−10 days with a mortality rate of 17−36%; however, PHF cases respond well to early medical intervention. Currently, the efficacy of inactivated whole-cell vaccines is debatable. Commercially available vaccines provide only limited or no protection (17), and this lack of protection has been clearly demonstrated in previous (19) and current study (Table 3). The inclusion of immune-protective antigens derived from contemporary *Neorickettsia* species including *N. findlayensis* could improve the efficacy of vaccines against PHF.
