# Supplementary material for: Real-Time PCR Differential Detection of Neorickettsia findlayensis and N. risticii in Cases of Potomac Horse Fever
Source: J Clin Microbiol. 2022 Jun 13;60(7):e00250-22. doi: 10.1128/jcm.00250-22 (PMC9297838; doi:10.1128/jcm.00250-22)
Supplement: Supplemental file 1 — Fig. S1 to S3. Download jcm.00250-22-s0001.pdf, PDF file, 0.7 MB [file jcm.00250-22-s0001.pdf]

Supplementary Data for

Real-Time PCR Differential Detection of *Neorickettsia findlayensis* and *N. ristícii* in Cases of Potomac Horse Fever

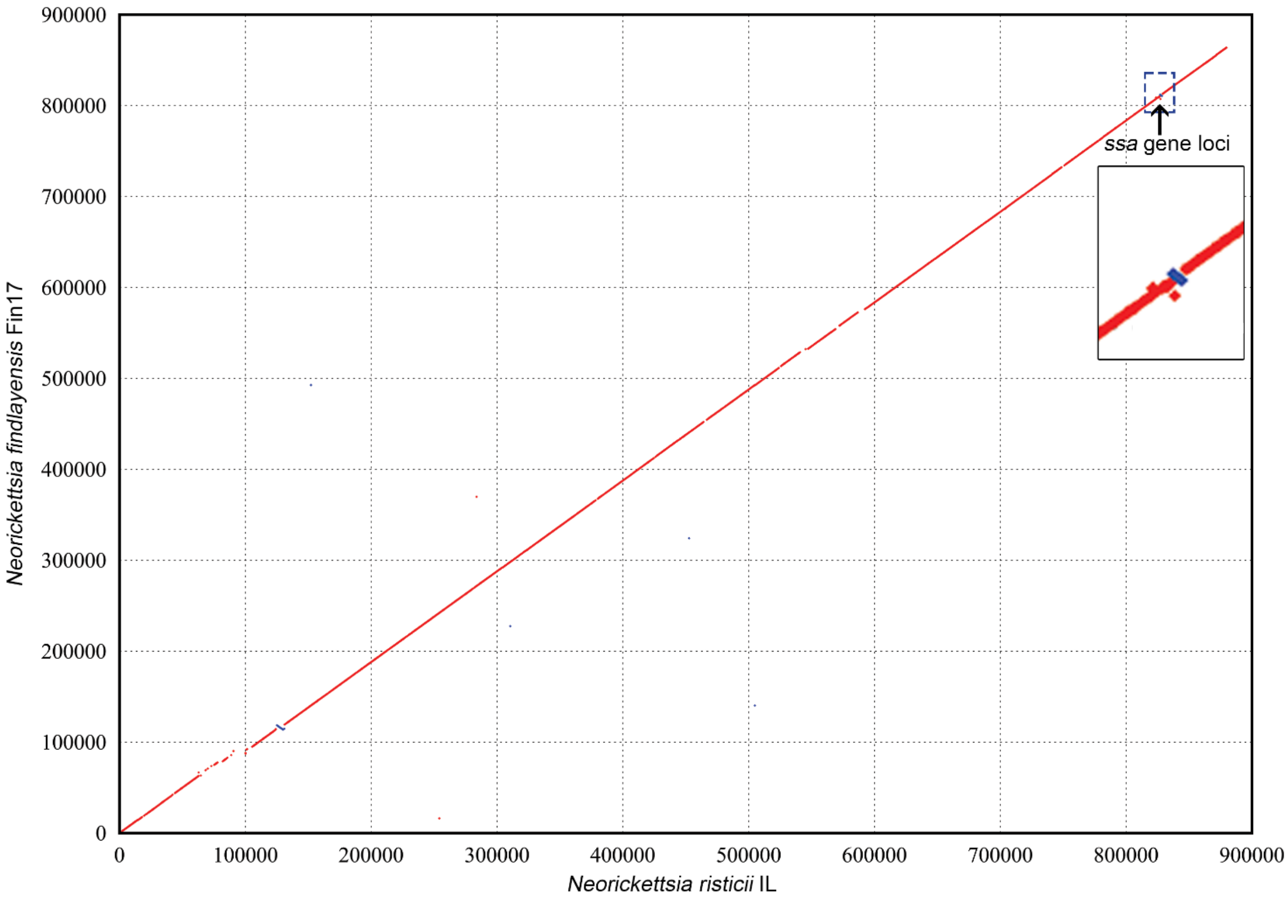

FIG. S1. Dot-plot of synteny alignments between *N. findlayensis* Fin17 and *N. risticii* IL.

The entire genome sequences of *N. findlayensis* Fin17 and *N. risticii* IL were aligned using MUMmer 3 program with default parameters. Each dot represents a pair of probable sequence fragments defined as reciprocal BLAST best hits with E-value < 0.001 (red, sequences match with the forward strand; blue, sequences match with the reverse complementary strand). Numbers represent base pairs, and the boxed region with dotted line indicates genomic loci encoding *ssa1* - *ssa3* genes (enlarged 5× in the inset).

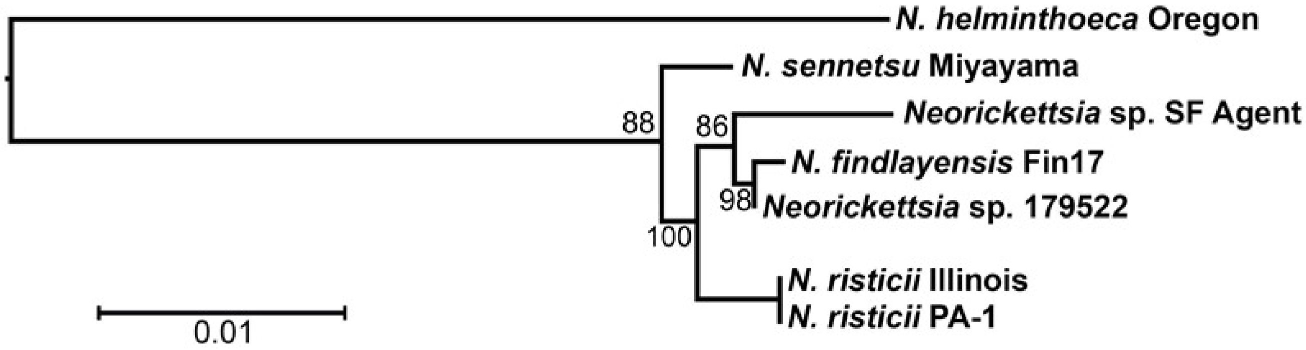

FIG. S2. Phylogenetic analysis of 16S rRNA genes of *Neorickettsia* species.
Sequences of *Neorickettsia* spp. 16S rRNA genes were aligned using the Clustal Omega algorithm, and the phylogenetic tree was constructed with MegAlign Pro (DNAStar Lasergene 17). Bootstrap values were calculated by maximum likelihood using RAxML options. The bar indicates nucleotide sequence distances: the number of nucleotide substitutions per site. NCBI accession numbers and/or locus tags of 16S rRNA genes: *N. risticii* PA-1: AF380257.1; *N. risticii* Illinois: NC_013009.1 (NRI_RS00185); *N. findlayensis* Fin17: NZ_CP047224.1 (GP480_RS00180); *N. helminthoeca* Oregon: NZ_CP007481.1 (NHE_RS00195); *Neorickettsia* sp. 179522: NZ_LNGI01000001.1 (AS219_RS00185); *N. sennetsu* Miyayama: NC_007798.1 (NSE_RS00200); and *Neorickettsia* sp. SF agent: U34280.1.

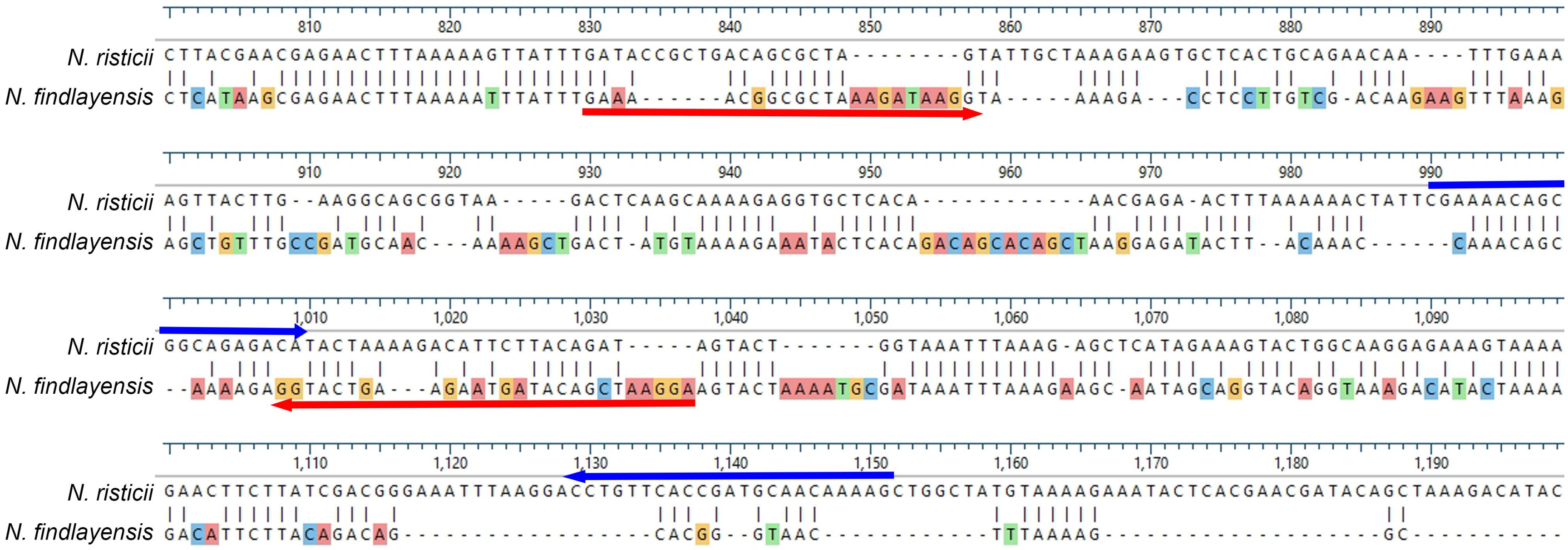

FIG. S3. ***Neorickettsia*** **species-specific qPCR primers for** ***ssa2*** **gene.**

N. findlayensis and *N. risticii ssa2* genes were aligned using the Clustal Omega algorithm by MegAlign Pro (DNAStar Lasergene 17), and primers were designed to amplify *N. risticii*- or *N. findlayensis*-specific *ssa2* genes. To ensure the specificity for species-specific *ssa2* genes, each primer was designed to contain at least 5 unique nucleotides at the last 10 nucleotides of the primer’s 3'-end based on the aligned sequences. *N. findlayensis*-specific primers (Nfin_ssa2F and Nfin_ssa2R) are indicated by red arrows below sequence alignment, and *N. risticii*-specific primers (Nris_ssa2F and Nris_ssa2R) are indicated by blue arrows above sequence alignments.
